# Evolutionary decoupling of morphological and behavioural antipredator responses and their hormonal basis across anuran species

**DOI:** 10.1098/rspb.2026.0887

**Published:** 2026-10-28

**Authors:** Rafael Rico-Millan, H. Christoph Liedtke, Lee Hyeun-Ji, Daniel R. Buchholz, Ivan Gomez-Mestre

**Affiliations:** ^1^ Ecology and Evolution, Estación Biológica de Doñana, Seville, Spain; ^2^ Naturschutzbund Deutschland eV, Berlin, Germany; ^3^ Biological Sciences, University of Cincinnati, Cincinnati, OH, USA

**Keywords:** phenotypic plasticity, stress response, predation risk, inducible defences, trait evolution

## Abstract

Adaptive plasticity allows organisms to adjust their phenotypes to environmental heterogeneity. Defensive responses to predation risk exemplify such plasticity, which, in the case of amphibian larvae, can involve changes in morphology, behaviour, physiology and life histories. These defences are hypothesized to be mediated by the glucocorticoid hormone corticosterone (CORT), but empirical support across species is inconsistent. We experimentally tested the generality of CORT involvement in predator-induced plasticity across larvae from 12 phylogenetically distant anuran genera, exposing them to either predation cues or different concentrations of exogenous CORT and comparing their responses. We measured changes in morphology, activity and life‐history traits and quantified endogenous CORT levels. Species showed pronounced interspecific variation in morphological and behavioural plasticity. Perceived predation risk did not consistently elevate endogenous CORT concentrations across species. Moreover, predator‐induced changes in body shape were generally distinct from, and even opposite to those elicited by exogenous CORT, indicating that CORT is not a direct regulator of anti‐predator defences in amphibian larvae. Nonetheless, we found evidence for an indirect association between CORT and antipredator responses such that species with higher baseline CORT levels tend to have faster developmental rates, greater CORT response to predation cues but reduced morphological responses and *vice versa*.

## 1. Introduction

Organisms are exposed to spatial and temporal environmental variation and have evolved sensory systems to assess the environment and generate appropriate phenotypic responses. In animals, the sensorial stimuli activate neuroendocrine responses that result in adjustments in behaviour, physiology, morphology and/or life history traits [1,2]. In vertebrates, the hypothalamus–pituitary–adrenal/interrenal (HPA/HPI) axis is key in regulating such organismal responses to environmental changes, partly through changes in the level of circulating glucocorticoids [3,4]. Glucocorticoids can have complex effects on energy allocation, metabolism, developmental and growth rates, reproduction and behaviour [5,6]; they are involved in the expression of up to 10% of genes, and they also interact synergistically with other hormones like thyroid hormone, greatly impacting vertebrate development [7,8]. While glucocorticoid-induced responses to short-term risks can be beneficial, the prolonged production of glucocorticoids (i.e. chronic stress) can result in non-adaptive effects, such as immune system suppression, depletion of energy reserves or increased mortality risk [9–12].

Glucocorticoids are key determinants of ontogenetic trajectories and survival, and yet few studies (e.g. [13–15]) have conducted standardized tests in a phylogenetic framework to associate variation in glucocorticoids with fundamental aspects of the biology of species, like developmental and growth rates, or the ability to plastically modulate these in response to environmental challenges. Common garden experiments conducted across multiple species are needed to understand the evolution of mechanisms of plastic responses to environmental stressors, but are logistically challenging and consequently still scarce (but see [16–19]). A near-universal and important environmental stressor is predation risk. However, the extent of this risk is not constant, varying in intensity across time and space [20,21]. Animals often detect predation risk through a variety of acoustic, visual and chemical cues, and shift their phenotype accordingly [22,23]. Upon detection of predator cues, prey can display a wide range of defences, from changes in behaviour and activity patterns to altered metabolic rates or defensive morphotypes [22,24–26]. Behavioural and morphological antipredator responses may be linked through the same regulatory mechanisms, and species vary in the prevalence or resource allocation of one type of response compared with the other [27,28].

Amphibian larvae are ideal for studying inducible responses to predation risk. In the presence of predators, tadpoles can reduce the time spent foraging and increase the use of shelters [29,30]. Moreover, they can change morphology by expressing rounder bodies, shorter tail length, increased tail depth and increased distal tail pigmentation, or adjust their developmental rate to accelerate or delay metamorphosis [31–33]. These responses vary widely across species, showing different evolutionary rates [18] and even affecting diversification rates [34]. Nevertheless, despite numerous studies on inducible defences in amphibian larvae, their endocrine regulation, and in particular the role of corticosterone (CORT) in generating these antipredator defences remains inconclusive. CORT is the main glucocorticoid in amphibians regulating metabolism, mobilization of energy reserves and stress responses, and together with thyroid hormone is the main driver of development and metamorphosis in anurans [8,35–37]. Predator presence induces increased CORT levels in larvae of some species [38,39]. However, other species do not change CORT levels when exposed to predation cues [40], and some may even reduce their CORT levels in the presence of predators [41–43]. Moreover, other stressors that also elicit changes in CORT secretion are known to induce dissimilar phenotypic effects in tadpoles to those caused by predator cues [44]. For instance, both high larval density and low food availability are known to increase CORT [10,45], but induce morphological changes opposite to those induced by predator cues [44,46]. Moreover, increased CORT is known to result in higher metabolic rate [36,47], whereas predator presence may cause a reduction in metabolic activity [42,48], suggesting that predator-induced changes in metabolism could be more congruent with decreased CORT levels than with increased ones. Moreover, baseline CORT is known to vary with latitude and altitude in amphibians [49], and across populations depending on patterns of water acidity [49] and chronic exposure to predator presence [50]. Consequently, amphibian species with divergent developmental rates and specialized in very different habitats are likely to show differences not only in baseline CORT, but also in its production in response to predator presence and its phenotypic consequences.

Here we take an experimental comparative approach to compare the role of CORT in mediating antipredator responses in larvae across anurans that vary greatly in body size, developmental rate and phylogenetic histories. We measure changes in endogenous CORT and quantify behavioural and morphological responses to predation cues for 12 anuran genera through standardized manipulation of predation cues or exogenous CORT. We test the hypothesis that the effect of exogenous CORT on activity rate and tadpole morphology mimics predator-induced changes. We also test whether among-species variation in baseline CORT levels is associated with developmental rate and with the degree of sensitivity to predation cues. We expect fast-developing species, breeding in ephemeral ponds (with lower predation pressures), to show increased baseline CORT, but reduced antipredator responses. Finally, we test the strength of the correlation between magnitude of behavioural responses and magnitude of the morphological responses across species.

## 2. Methods

### (a) Animal husbandry and experimental set-up

We studied 12 species, each from a different genus, representing nine families: *Alytes cisternasii*, *Discoglossus galganoi*, *Xenopus laevis*, *Spea multiplicata*, *Pelodytes ibericus*, *Pelobates cultripes*, *Hyla meridionalis*, *Bufo spinosus*, *Epidalea calamita*, *Pelophylax perezi*, *Rana temporaria* and *Lithobates sylvaticus*. Larvae from these species vary greatly in size, duration of the larval period and habitat preference, with species breeding in small, ephemeral ponds showing fast development, small size at metamorphosis and a lower exposure to predation risk [33,51]. Egg clutches or tadpoles from each species were collected between February 2020 and March 2023 from their native ranges in Spain and North America, except for *X. laevis* and *S. multiplicata*, which were obtained from captive breeding programmes (supplementary material, table S1). For *D. galganoi, X. laevis, S. multiplicata, P. ibericus, P. cultripes, B. spinosus, E. calamita, R. temporaria* and *L. sylvaticus*, we collected *ca* 150 eggs from three to five clutches for each species in order to avoid parental bias and ensure a minimal representation of various sibships. In the case of *A. cisternasii, H. meridionalis* and *P. perezi*, we could not use egg clutches either to avoid capturing or disturbing adults or owing to increased difficulty of finding clutches, so we collected larvae in early developmental stages (Gosner stage ≤25; [52]) from different parts of each pond to maximize the odds of sampling different sibships.

Clutches and tadpoles were transported within 3 days of their collection to the climatic chambers at Doñana Biological Station (Seville, Spain) and housed at a constant 20°C and 12 : 12 h light–dark cycle. We kept clutches well aerated in shallow trays with carbon-filtered dechlorinated tap water. Once embryos hatched and reached the free-feeding larval stage (Gosner stage 25), we haphazardly individualized 128 tadpoles per species in 1 l tapered plastic containers (13 cm height, 11 cm top diameter and 9 cm base) filled with 800 ml of water. We fed the tadpoles ad libitum with a mixture of ground rabbit chow and boiled spinach, renewing the water twice a week.

Once tadpoles reached Gosner stages 28–29, we simultaneously set up two complementary 10-day experiments aiming to test the effects of exogenous CORT and waterborne predation cues on morphology, activity and endogenous CORT levels. For the CORT experiment, we exposed a subset of the individualized tadpoles to 50 or 100 nM exogenous CORT concentrations, obtained by adding 100 µl of either 0.4 or 0.8 mM CORT stock solutions, respectively, to the container. As CORT was dissolved in ethanol, controls received 100 µl of ethanol. We renewed the water and the corresponding treatment daily to overcome the natural decay of CORT. We set up 24 replicates for each treatment per species.

In the second experiment, we exposed another subset of tadpoles of each species to the presence or absence of predation cues. To generate these cues, between five and 15 dragonfly larvae (*Anax* spp.) or diving beetle larvae (*Dytiscus*µ) were housed individually in 1 l aquaria. Owing to differences in tadpole species’ phenology and the availability of predator species, we could not standardize the number of individual predators and the composition of *Anax* and *Dytiscus* used to obtain the cue across all amphibian species. Seven species were exposed to a mix of *Anax* and *Dytiscus*, whereas five species were only exposed to *Anax* (supplementary material, table S1). Moreover, although all these anuran species are exposed to predators of these same genera in their native regions, some of them are not sympatric with the particular predator species that we used, which could affect anti-predator responses of the tadpoles [53]. To ensure that we provided a reliable predation cue to the experimental animals [54], each individual predator was fed one experiment-specific tadpole species, so that the water in their aquaria would contain predator kairomones, along with alarm cues and digestion cues from consumed conspecific tadpoles. We combined water from at least two predator containers to reach approximately 500 ml for each of the tadpole species, which was then vacuum-filtered using a 43–48 µm filter (1300/80 qualitative filter paper, FILTER-LAB). We added 10 ml of water containing predation cues to 28 tadpoles per species and 10 ml of dechlorinated water to the same number of control tadpoles. Identically to the exogenous CORT experiment, we renewed the water and hormonal treatments daily for 10 days.

On the tenth day, we conducted behavioural assays to measure activity rate. We arranged groups of 16 individual containers on the floor over a white background, and recorded them from above for 30 min, after a 5 min acclimation period, using a digital video camera (Handycam HDR SR11, Sony). Filming took place between 09.00 and 16.00 for all species, to film only diurnal activity. Immediately after, we took lateral photographs of tadpoles in a Plexiglass photo booth with a Nikon D50 camera and Tamron 90 mm macro lens, for geometric morphometric analysis (see below). Five tadpoles per treatment from the exogenous CORT experiment and 14 tadpoles per treatment from the predation experiment were then euthanized by immersion in a buffered MS-222 solution (ethyl 3-aminobenzoate methanesulfonate, Sigma-Aldrich) and snap-frozen in liquid nitrogen for determination of CORT levels.

The remaining tadpoles from the predator experiment were returned to their containers. We continued applying the treatments until they reached Gosner stage 42 (forelimb emergence). At this point, we reduced the water depth to approximately 2 cm and added soaked tissue paper to provide refuge until metamorphosis completion (Gosner stage 46, tail resorption).

### (b) Determination of endogenous corticosterone concentrations

Endogenous CORT concentrations were assayed using DetectX CORT ELISA kits (K014-H5, Arbour Assays). We extracted CORT from the entire tail, following the protocol for tissue extractions described in [55]. We assayed all samples in triplicate and prepared a new standard curve for each 96-well plate. We read the plates with an MB-580 automatic microplate reader (Heales) at 450 nm. We calculated the hormonal concentration with the MyAssays online tool (https://www.myassays.com/arbor-assays-corticosterone-enzyme-immunoassay-kit-improved-sensitivity.assay). Some CORT values were outside the standard curve's detectable range, resulting in the exclusion of one sample without any readable triplicate. Twenty-two out of 464 samples had detectable CORT concentrations but below the limit of quantification of the kit (LOQ = 14.35 pg ml^–1^). Although these measurements could have a higher estimation error, we opted to retain raw values (flagged as ‘<LOQ’), rather than excluding them from the analysis or imputing a fixed value like LOQ/2 that could bias the results [56]. All analyses described here and in the subsequent section were performed in R (v. 4.4.2) [57], unless stated otherwise. For triplicates with <30% coefficient of variation and an absolute difference between mean and median lower than 2.5 units, we kept the mean of the three measurements; otherwise, the most divergent triplicate was discarded. We corrected CORT concentrations by initial tail mass (pg CORT/mg tissue). The overall intraplate variation was 15.21% and average interplate variation was 24.88%. We performed linear models (CORT concentration ∼ treatment) separately for predation and exogenous CORT exposure experiments for each species, applying log transformation to the CORT concentration values to meet parametric assumptions. For the exogenous CORT experiment, we performed Tukey's *post hoc* tests.

### (c) Activity rate

We used Ethovision XT 16 video-tracking software (Noldus Information Technology) to quantify tadpole activity from the video recordings, after converting files to 720 p MP4 format. For the detection settings, we used the greyscale method, applying low pixel smoothing and a threshold of 4 units of colour per pixel to be considered as movement (i.e. a shift in tadpole position). We also set the background noise filter to level 1 to differentiate tadpoles from background and a sample rate of six measurements per second. After data acquisition, we selected the activity state option in the analysis profile section. We used a 0.05% total pixel change threshold between sequential images to differentiate active and inactive states, with a minimum state duration of 0.10 s. The output yielded the proportion of time that tadpoles spent active. After performing an arcsine transformation of the data to meet parametric assumptions, we conducted species-specific linear models to test for differences in activity rate due either to exogenous CORT or to predator cues, followed by Tukey's *post hoc* tests for the CORT experiment.

### (d) Geometric morphometrics

To analyse morphological variation of tadpoles in response to predators or exogenous CORT, we digitized the lateral photos using a series of nine landmarks and 24 semilandmarks, using tpsDig2 v. 2.31 [58]. The landmark distribution provided a comprehensive shape representation of the body, tail muscle and upper tail fin (supplementary material, figure S1). The bottom tail fin was excluded because tadpoles usually rested on the bottom of the aquarium, resulting in an artificially straightened edge. The same landmark and semilandmark configurations were used for all species except *X. laevis*, for which we digitized semilandmarks on the ventral edge of the tail fin instead, as this species has only a marginal dorsal tail fin and tadpoles generally come to rest suspended in the water column. We used the function *unbend specimens* with a quadratic curve fitting in tpsUtil v. 1.47 to remove the potential effects of the bending of tadpoles’ bodies.

We used the R package *geomorph* v. 4.0.8 [59] for our morphometric analyses. To obtain comparable values of morphological change, we performed the subsequent analyses with the combined datasets of all species, except *X. laevis*, which, owing to its particular landmark configuration, was analysed separately following the same steps. We performed generalized Procrustes analysis using the bending energy criterion to optimize the position of the semilandmarks and align coordinates for all the species within the same morphospace. We fitted a linear model with a randomized residual permutation procedure using the *procD.lm* function to determine the effect of predation cues or exogenous CORT on tadpoles’ shape, using 1000 permutations. The model included species, the interaction between species and treatments, and the log-transformed centroid size as a covariate to control for allometry (coordinates ∼ treatment × species +  log(centroid size)). Afterwards, we performed *post hoc* pairwise comparisons among treatments. Additionally, we used the function *trajectory.analysis* to compare the trajectory of the morphological change induced by 100 nM CORT and predation cues relative to their controls for each species.

### (e) Larval period and weight at metamorphosis

We recorded tadpoles’ larval period as the number of days elapsed since the eggs were laid until they reached Gosner stage 46. For the species collected as tadpoles, we estimated the laying date based on the developmental stage of the larvae collected and the timing of previous rainfall events that triggered breeding [60,61]. Upon completion of metamorphosis, juveniles were weighed to the nearest 0.1 mg. To test the effect of predator cues on larval period and weight at metamorphosis, we conducted *t*-tests for each trait across species. We could not perform these analyses for *H. meridionalis*, owing to the low survival of tadpoles exposed to predation cues.

### (f) Phylogenetic comparative analyses

To test for trait associations across species in a phylogenetic comparative framework, we obtained a phylogenetic tree from VertLife (http://vertlife.org/phylosubsets/) for our study species. We downloaded 500 pseudo-posterior trees and then obtained a consensus tree using the package *phytools* v. 2.4-4 [62] in R. For estimating the degree of plasticity, we used the *effsize* package v. 0.8.1 [63] to calculate Hedges' *G* effect sizes in larval period and weight at metamorphosis for the predator-induced changes, and in CORT levels and activity for both predator- and exogenous CORT-induced changes. While different approaches exist for standardizing plasticity across species and traits (e.g. [18]), we chose Hedges' *G* to account for the large differences in trait means and s.d. across species and avoid possible bias due to small sample sizes [64]. Hedges' *G* was highly correlated with the logarithm of the response ratio (lnRR, [65]) for all traits except activity rate. For this trait, several species with low activity rate showed s.d. equal to or greater than the mean, and therefore Hedges' *G* was particularly suitable. For the morphological responses, we used the *Z-*value statistic provided by the pairwise comparison of the geometric morphometric analysis. We quantified the phylogenetic signal of the studied traits and traits’ plasticity using Blomberg's *K*, implemented in the *picante* package v. 1.8.2 [66]. Tests used 1000 randomizations. Furthermore, we tested trait associations with basal CORT level and larval period (i.e. basal trait values from individuals of control treatment in the predation experiment), as well as tests for trait integration between morphological and behavioural responses, fitting different phylogenetic linear models using several approaches for the error term (Brownian motion, Pagel's λ and Ornstein–Uhlenbeck), implemented in the *phylolm* package v. 2.6.5 [67]. Each model had 100 bootstrap replicates, and we used the lowest corrected Akaike information criterion (AICc) for selecting the best model fit (ΔAICc > 2 considered an improvement in model performance).

## 3. Results

For each species, we measured CORT concentration in the tail, as well as variation in activity rate, tadpole shape, larval period, and size at metamorphosis in response to different experimental treatments (figure 1). There was no discernible difference in effects between the two concentrations of exogenous CORT used, so only the results of the 100 nM concentration are discussed onwards. Analyses for both concentrations are presented in the supplementary material.

**Figure 1.**
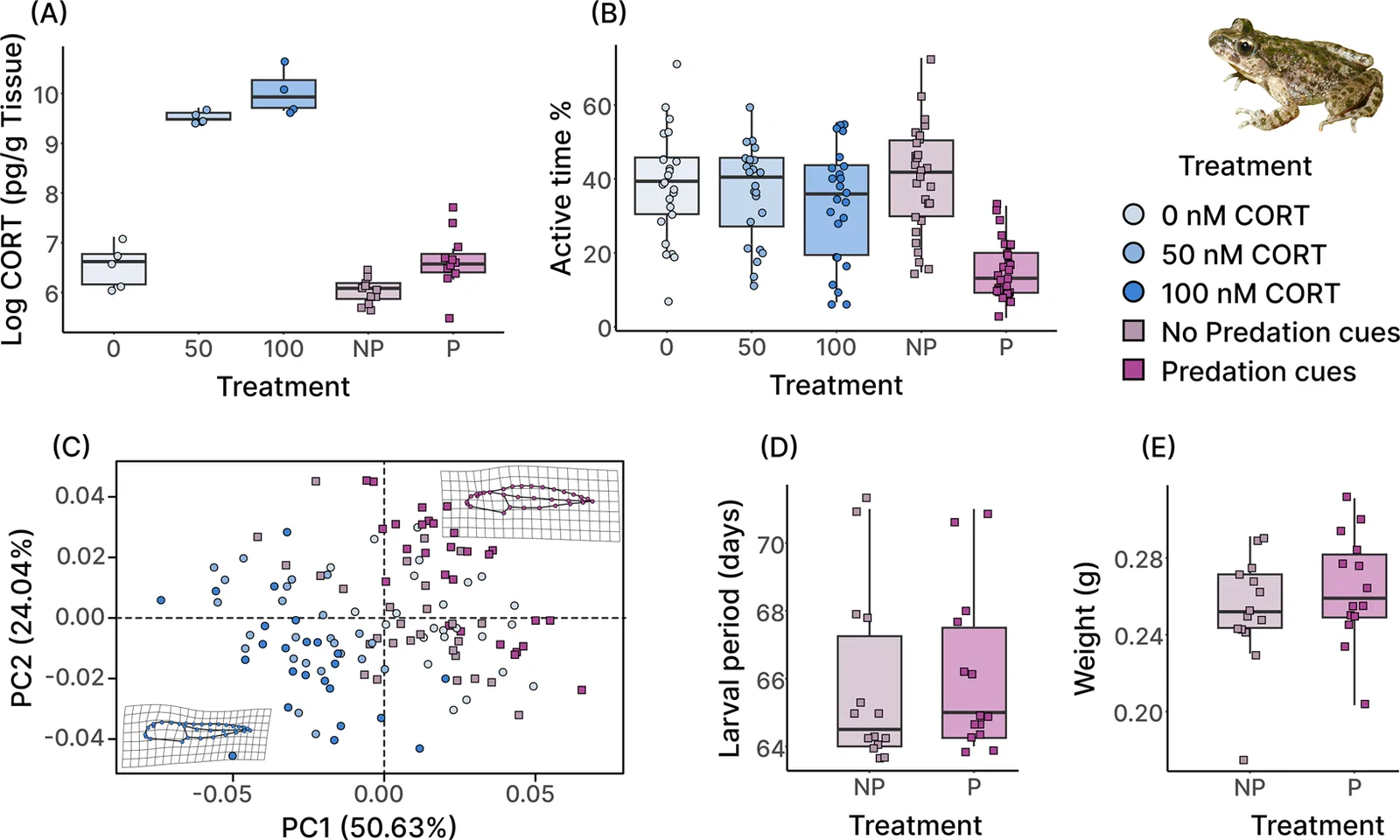
Example of data collected for each of 12 anuran species, exposed to exogenous corticosterone (CORT) concentrations (blue tones) or presence/absence of predation cues (P/NP, purple tones), illustrated here for *Pelodytes ibericus.* (A) Endogenous CORT level in the tail. (B) Proportion of time tadpoles spent moving during 30 minutes activity assays. (C) Principal component analysis on Procrustes shape coordinates and mean shape of tadpoles exposed to predation cues and to 100 nM CORT. (D) Larval period (days from egg-laying to Gosner stage 46). (E) Weight of juveniles after metamorphosis. Circles and squares represent individual values; boxplots indicate median, range and first and third quartiles.

### (a) Effects of exogenous corticosterone and predation risk on endogenous corticosterone levels

Tail CORT was significantly increased in all species by adding exogenous CORT to the water (figure 2B; supplementary material, tables S2 and S3). Exposure to predation cues also significantly increased CORT levels in *D. galganoi*, *P. ibericus* and *B. spinosus* (and marginally in *E. calamita*), and had no significant effect on CORT levels in the other species. Though not significantly so, in *P. cultripes* and *L. sylvaticus* the tendency was towards reduced CORT levels when exposed to predation cues (figure 2B; supplementary material, table S4).

**Figure 2.**
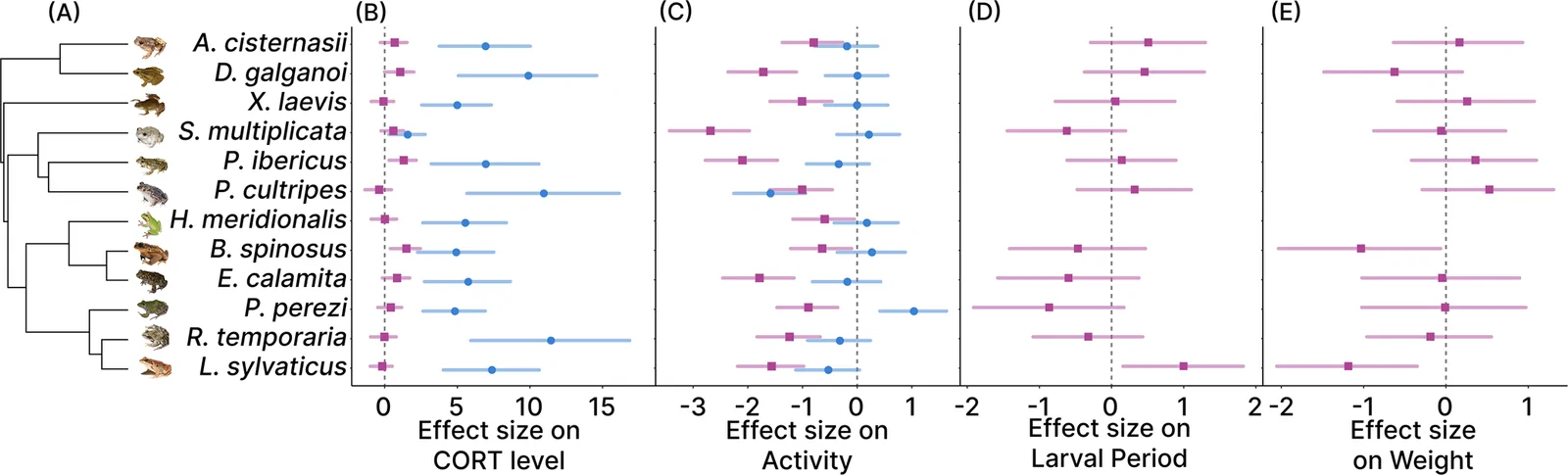
(A) Phylogenetic relationship among the studied species, and effects of predation cues (purple) and 100 nM exogenous corticosterone (CORT) (blue) on (B) endogenous CORT levels, (C) activity, (D) larval period and (E) weight at metamorphosis. Shown are Hedges’ *G* effect sizes with their standard errors.

### (b) Effects of exogenous corticosterone and predation risk on activity rate

Only tadpoles of *P. cultripes* and *P. perezi* showed significant changes in activity rate in response to exogenous CORT. Both concentrations of exogenous CORT induced decreased activity rate in *P. cultripes* and increased activity rate in *P. perezi* (figure 2C; supplementary material, tables S5 and S6). By contrast, all species consistently and significantly reduced their activity rates in response to predation cues (figure 2C; supplementary material, table S7).

### (c) Effects of exogenous corticosterone and predation risk on morphology

Exogenous CORT-induced morphological changes occurred in most species, except for *X. laevis* and *P. perezi*, but the effect was only marginally significant for *B. spinosus* and *E. calamita* (figure 3; supplementary material, tables S8 and S9). We observed significant morphological responses to predation cues in *A. cisternasii, S. multiplicata, P. ibericus, P. cultripes, H. meridionalis, R. temporaria* and *L. sylvaticus*, and marginally significant changes in *D. galganoi* (*p* < 0.1; figure 3; supplementary material, table S9). The trajectory analysis showed that exogenous CORT induced stronger morphological changes than predator presence in most species (figure 3; supplementary material, table S10). In general, tadpoles exposed to exogenous CORT produced a more elongated shape, with a shallower tail, particularly in the medial–posterior region of the fin, and more oval-shaped and conspicuous bodies compared with the control treatment (supplementary material, figure S2). When species responded to predation cues, the morphological change was usually in the opposite direction to exogenous CORT, but an important part of the morphological change occurred along a different axis of the morphospace (figure 3; supplementary material, table S10). Morphology of tadpoles exposed to predation cues was characterized by deeper tails, especially in the anterior region of the tail, with a more anterior insertion of the fin in the body (supplementary material, figure S2). *Xenopus laevis*, analysed separately owing to its unique landmark configuration, exhibited no significant morphological changes in response to either exogenous CORT or predation cues (supplementary material, tables S11 and S12).

**Figure 3.**
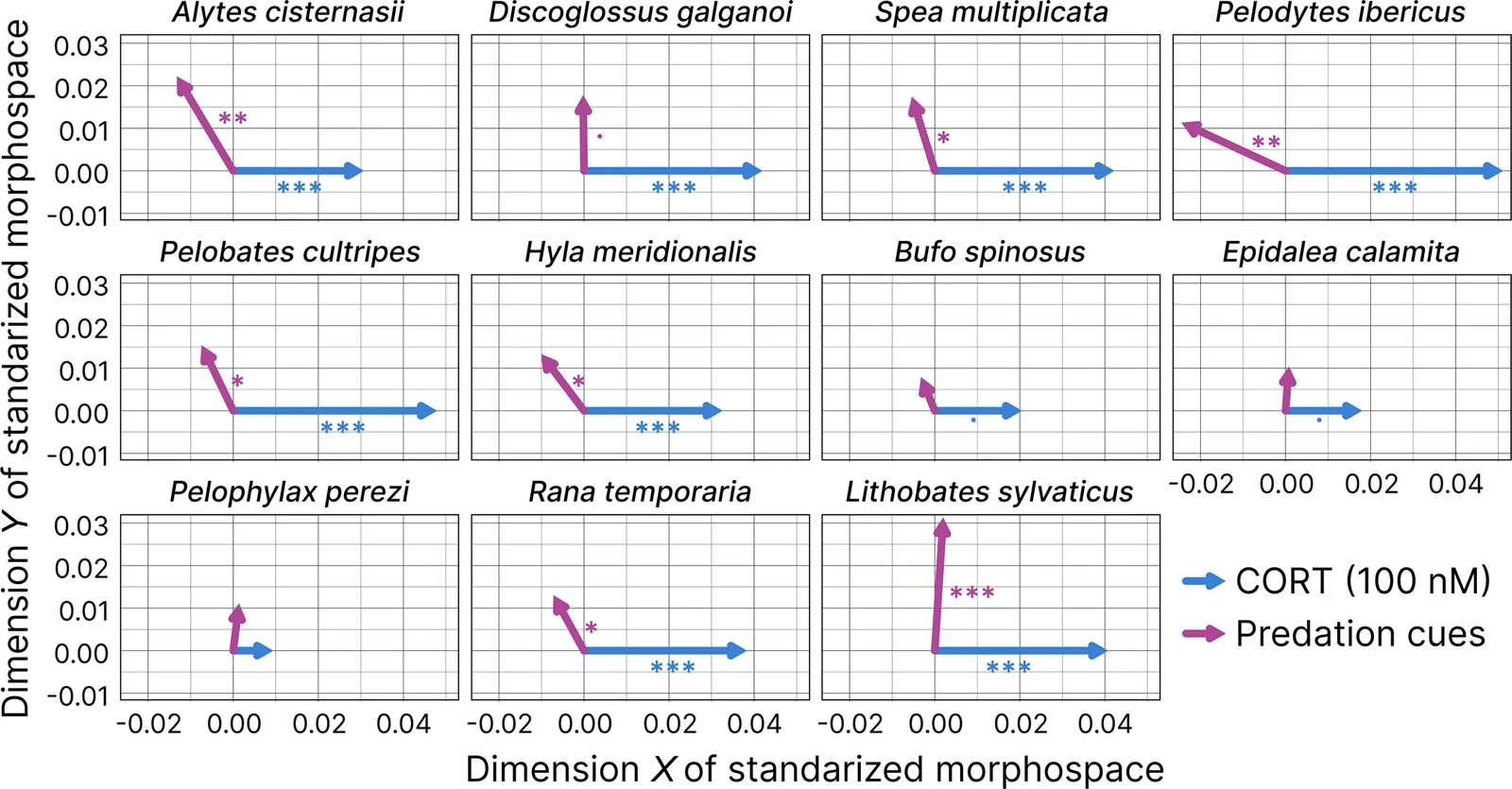
Vectors of morphological change induced by 100 nM corticosterone (CORT) (blue) or predation cues (purple). The vector for 100 nM CORT was oriented parallel to the *X*-axis as reference for comparing across species. Vector lengths and angles indicate the magnitude and direction, respectively, of morphological change. Significance levels: ***p < 0.001, **p < 0.01, *p < 0.05, *p* < 0.1.

### (d) Effects of predation risk on larval period and size at metamorphosis

Larval period was unaffected by exposure to predation cues, except in *L. sylvaticus* (figure 2D; supplementary material, table S13), which increased its larval period by 5.99 days (*t*-value = 2.604, *p* = 0.016). Weight at metamorphosis was also largely unaffected, except for juveniles of *L. sylvaticus* and *B. spinosus* (weight difference = –0.042, *t*-value = –3.156, *p* = 0.004; weight difference = –0.010, *t*-value = –2.362, *p* = 0.029), which metamorphosed at lower weights than conspecific animals from the control treatment (figure 2E; supplementary material, table S14). *Hyla meridionalis* was not included in the analysis because all tadpoles exposed to predation cues died before completing metamorphosis.

### (e) Comparative phylogenetic analyses of corticosterone variation and phenotypic responses

We found marginally significant phylogenetic signal for the baseline activity of tadpoles (Blomberg's *K* = 0.728, *p* = 0.067). All other traits and their plasticity (effect sizes) did not show significant phylogenetic signal (supplementary material, table S15).

Baseline CORT levels (i.e. CORT levels of control individuals in the predation experiment) were inversely correlated with larval period across species, so that species with shorter larval periods had higher levels of CORT (adjusted *R*^2^ = 0.247; slope (*b*) = 16.731, *t-*value = –2.148, *p* = 0.057; figure 4A). Mass at metamorphosis also showed a negative correlation with baseline CORT levels (adjusted *R*^2^ = 0.377; *b* = –0.652, *t*-value = –2.768, *p* = 0.020; figure 4B). However, we did not find a relationship between baseline activity rate (i.e. activity rate of control tadpoles) and the baseline CORT level (adjusted *R*^2^ = –0.0243; *b* = –0.187, *t*-value = –0.536, *p* = 0.410; figure 4C).

**Figure 4.**
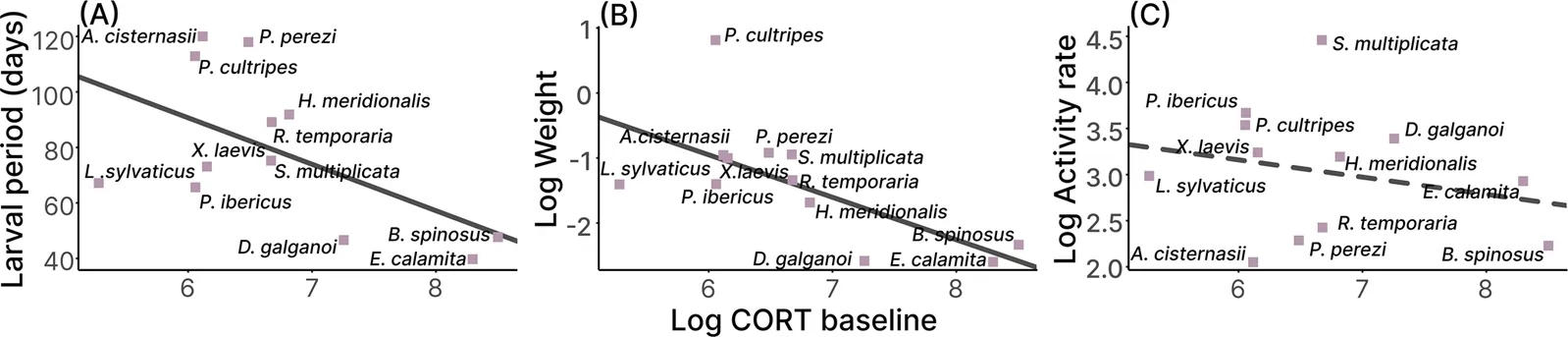
Relationships of baseline corticosterone (CORT) (predation experiment control groups) with (A) larval period, (B) weight at metamorphosis and (C) activity across species. Solid and dashed regression lines indicate significant and non-significant relationships respectively (*p*-value < 0.05 as significance threshold).

Baseline CORT levels and larval period were also significantly correlated with the magnitude of responses (i.e. plasticity) to predation cues in most traits (but not all) (figure 5). The effect size for endogenous CORT changes was positively correlated with baseline CORT level and negatively correlated with larval period (albeit marginally so), so that species with higher baseline CORT levels and shorter larval periods increased CORT more when exposed to predation cues (table S16; figure 5A,B). Changes in activity rate were not correlated with baseline CORT levels, although they were correlated with larval period, so that species with shorter larval periods showed the greatest reduction in activity in response to predation cues (table S16; figure 5C,D). Morphological change was inversely correlated with baseline CORT levels, meaning that the greatest morphological induction was seen in species with the lowest baseline levels of CORT (table S16; figure 5E). Larval period plasticity was negatively correlated with baseline CORT level, whereas the effect on juvenile body mass did not show any correlation with CORT level or larval period (table S16; supplementary material, figure S3). However, it is worth noting that for these two traits, only a few species showed significant differences in response to predation risk.

**Figure 5.**
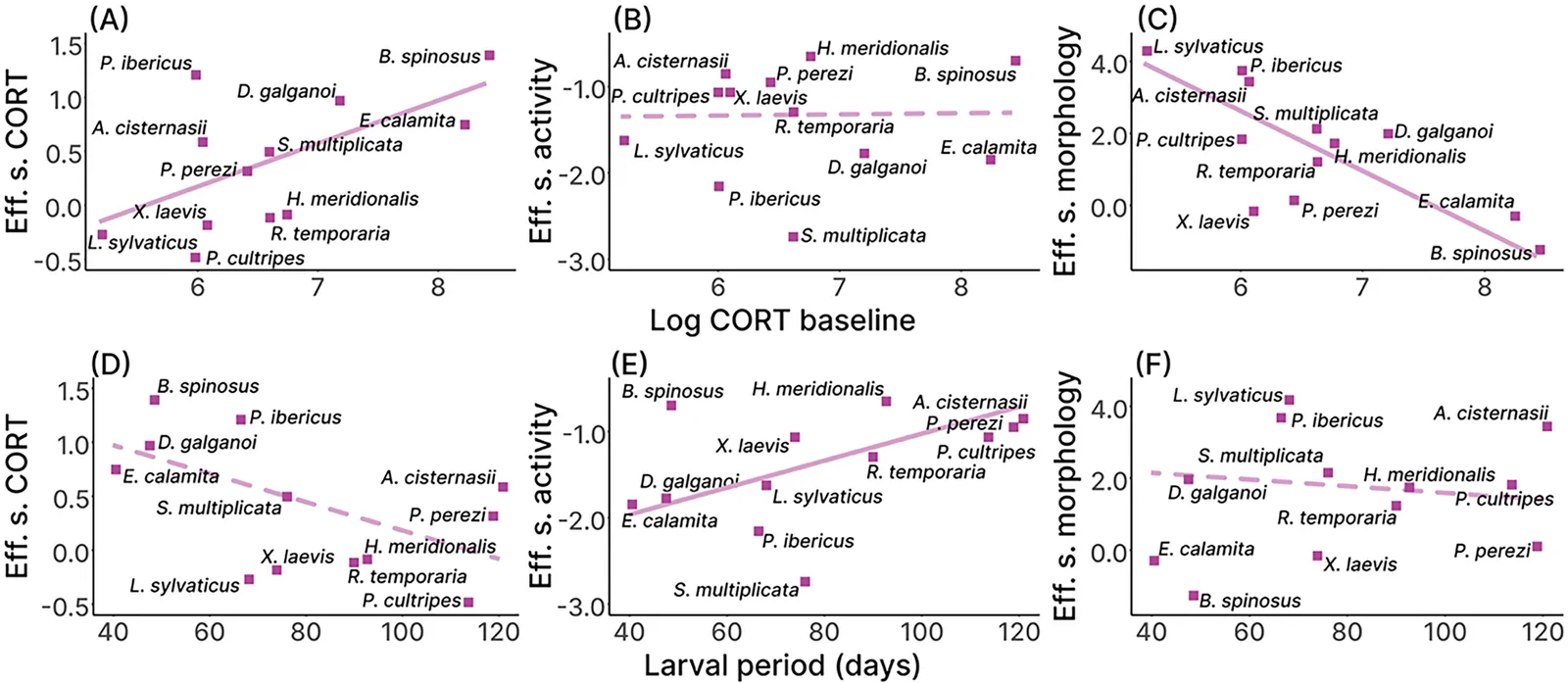
Relationship of endogenous corticosterone (CORT) baseline levels and larval period length with the effect size (Eff. s.) of exposure to predation cues on: (A,D) endogenous CORT levels, (B,E) activity and (C,F) morphology. The effect sizes are Hedges’ *G*-values except for morphology, where *Z*-values from the model are used instead. Solid and dashed regression lines indicate significant and non-significant relationships, respectively.

The magnitude of the endogenous CORT response to predation cues was not associated with the magnitude of phenotypic shift in any of the studied traits (supplementary material, table S17). The magnitude of change in CORT level of different species when exposed to exogenous CORT was associated with the magnitude of the change induced in activity rate, but not with the effect size on morphology (supplementary material, figure S4 and table S17).

In response to predation cues, we observed no association between behavioural responses (i.e. reduced activity rate) and morphological responses (adjusted *R*^2^ = 0.065; *b* = −0.946, *t*-value = −1.326, *p* = 0.214; supplementary material, figure S5A). However, when exposed to exogenous CORT, species showing a stronger reduction in activity rate also tended to produce a greater morphological change (adjusted *R*^2^ = 0.231; *b* = −1.657, *t*-value = −2.076, *p* = 0.065; supplementary material, figure S5B), although this effect was mostly driven by *P. cultripes* and *P. perezi*, showing opposite patterns.

## 4. Discussion

Through standardized experimental testing and comparative analyses, we assessed changes in behaviour, morphology and CORT levels in response to predation cues across 12 anuran genera, and compared these with changes induced by exogenous CORT.

### (a) Behavioural, morphological and developmental responses to predation cues

We found highly consistent behavioural responses to predation cues across the studied species: all species reduced their activity rate in response to predation cues, even though the magnitude of the response varied across species. Despite our effort to standardize the preparation of predator cues across species, not all species could be exposed to a mixture of both *Anax* and *Dysticus* larvae, and although all predators were fed a conspecific tadpole prior to the assays, tadpoles differed in biomass, which could also have introduced some noise to the experiment [68]. Nevertheless, the consistent behavioural response across species, congruent with most studies on antipredator behaviour in anurans, suggests that all species perceived the combined predator kairomones and conspecific alarm cues as a potential risk. Most species (eight out of 12) also showed morphological responses to predation cues, adopting rounder bodies, increased depth of the anterior part of the tail, and a more anterior insertion of the tail fin in the body, also congruent with previous literature [31,69]. Behavioural and morphological responses may be co-expressed in some species [70], but we did not find evidence for this association in a phylogenetic context. Therefore, species showing a strong behavioural response to predators did not necessarily also show a strong morphological response and *vice versa*. The fact that all species reduced activity in response to predation cues, whereas morphological responses were less consistent across species, supports the notion that behavioural antipredator responses may have been the primary antipredator inducible defence [71,72]. Morphological responses, in turn, may have originated as by-products of altered hormonal levels and then been co-opted as antipredator responses in cases where the change in shape also contributed to reduced predation risk [27,71]. Indeed, experiments assessing the relative effect of predator-induced behaviour and morphology on survival indicate that morphological responses *per se* may not further improve survival [28,73]. Behavioural responses are usually also highly reversible, allowing a better phenotypic adjustment under temporal variability in predation risk than morphological responses [74,75]. However, behavioural and morphological defences may complement each other in complex environments since the efficiency of one or the other may be highly context-dependent [28,76]. For instance, under increased tadpole density, behavioural responses may be suppressed as tadpoles find it unsustainable to reduce their activity rate in a scenario of intense competition [77], and also not all inducible responses (whether behavioural or morphological) may be equally effective against different types of predators [32,78]. By contrast to the above effects, predation cues had little influence on growth and developmental rate across species. Exceptions were *B. spinosus* and *L. sylvaticus*, both showing a reduction in weight at the end of metamorphosis, and only *L. sylvaticus* showing a slight increase in larval period.

### (b) Hormonal responses

In response to predation cues, two species had a tendency towards a reduction in CORT, and three species had significantly increased CORT levels. The decreasing trend seen here in *P. cultripes* is corroborated by previous reports [42,43]. This trend has also been found for *Hylarana indica* after chronic exposure to predators [79]. A reduction in CORT is also consistent with reports of predation cues inducing reduced metabolic rate in other amphibians and poeciliid fish [80,81], a common consequence of decreased glucocorticoid levels across vertebrates [47,82,83], though decreased activity leading to decreased food intake may also account for reduced metabolic rate. The three species showing increased CORT in response to predation cues have the shortest larval periods. Previous work on tadpoles reflects substantial variation in induced CORT levels across species as well as variable temporal dynamics in the hormonal levels induced. *Lithobates sylvaticus* tadpoles have been shown to decrease CORT levels when first exposed to predation cues [84], but then increase CORT after 4 days of exposure [39]. Interestingly, other populations of this species show the opposite pattern, producing an acute CORT increase in response to the presence of predators (10–20 min after) that eventually dwindled and resulted in lower CORT levels after three weeks of chronic exposure to predation cues compared with naive tadpoles [85]. Within-species geographical variation in the degree of CORT response to predator presence is also apparent for Swedish populations of *R. temporaria* [50]. The high sensitivity of CORT production to environmental conditions and non-standardized experimental procedures across studies impede a straightforward interpretation of the relationship between predation cues and CORT production. Under our standardized conditions, the effect of predation cues on CORT levels was variable across species in magnitude and direction.

### (c) Lack of causal relationship between induced antipredator responses and corticosterone levels

We found no evidence of a causal relationship between CORT and antipredator responses. Predator-induced changes in activity rate and morphology were not associated with shifts in CORT levels. Furthermore, the addition of exogenous CORT did not generally have an effect on activity rate. Regarding morphology, previous studies suggest a direct causal link between increased CORT concentration and tail depth in *L. sylvaticus* [39,86], although those results were based on isolated linear measurements of morphological traits (tail depth, size and muscle depth) and were corrected either by total length or by mean mass. Experimental treatments could potentially alter overall body proportions nonlinearly, and therefore the perception of changes in tail depth may also have been influenced by changes in total size. By using a geometric morphometric approach, we analysed the complete landmark geometry of tadpoles simultaneously, allowing us to overcome the confounding effects of allometry. We found that wood frog (*L. sylvaticus*) tadpoles, as the other species studied, responded differently to predation cues as compared with experimentally increased CORT levels. Experimentally increased CORT induced a great shift in tadpole morphology, but did so in largely independent directions of the morphospace from the changes induced by predation cues. Therefore, although both predation cues and CORT have an effect on body shape, they affect fundamentally different aspects of the morphology, and we cannot conclude that the evolution of predator-induced morphologies has been achieved through increased CORT signalling. In fact, CORT-induced morphotypes (oval bodies, shallower tails and more posterior insertion of the tail fin) are analogous to morphological changes typically associated with increased competition, thought to be antagonistic to predator-induced changes [44,46,87]. This link between increased CORT and the expression of a typical competition-induced morphotype is not surprising because high competition results in low food availability and crowding, factors known to increase CORT in amphibian larvae [37]. Therefore, we can conclude that chronic exposure to predators may result in an increase or decrease in CORT levels in different species, and that such variation in CORT levels is not necessarily causally linked with the deployment of antipredator morphological defences. However, it is also noteworthy that species showing minimal antipredator morphological responses (e.g. *B. spinosus*, *E. calamita* and *P. perezi*) also show modest morphological responses to exogenous CORT (figure 3). Further work on the mechanisms determining tail and body shape across species is needed to understand why some species are highly sensitive and yet others quite refractory towards altering their body shape in response to predation cues.

### (d) Evolutionary patterns of divergence in plastic responses

Despite the apparent lack of direct regulation of antipredator responses by CORT, we observed strong associations between baseline CORT levels and fundamental aspects of larval biology that suggest an indirect link between CORT and the species’ capacity for plasticity. First, we found an association between the level of baseline CORT and developmental rate, such that fast-developing species tended to have higher CORT levels. We acknowledge that the standardized temperature used in our experiment could affect the developmental rate of species that usually breed during colder or hotter seasons in the year, but we obtained a similar correlation using larval period data extracted from the literature (supplementary material, figure S6). This association between CORT level and larval period was previously detected within spadefoot toads [36], and our results suggest that it may be generalized more broadly across anurans.

Moreover, species with high baseline CORT levels tended to show comparatively muted morphological responses to predators, even if they experienced large increases in endogenous CORT when exposed to predation cues. Concurrently, there was no direct association between baseline CORT and activity rate reduction in response to predators, although species with shorter larval periods had a stronger reduction in activity. These observations may constitute adaptations to different habitats where hydroperiod and predation risk exert selective pressures resulting in different strategies. Energy allocation to development, driven by higher CORT levels in fast developers, may constrain morphological responses to predation, whereas reversible and less costly behavioural responses, probably controlled by a different mechanism, are present in all species. However, the relationship between glucocorticoids and life-history traits is complex, and we cannot discard that high CORT levels and reduced morphological plasticity could be independent adaptations to ephemeral habitats.

## 5. Conclusions

We found that, across 12 anuran genera in nine families, all species consistently reduced their activity rate in response to predators, but showed more disparity in their morphological responses. Induced morphological shifts range from very minor to conspicuous increases in tail depth, anterior insertion of the tail on the body and rounder bodies. Induced behavioural and morphological antipredator responses, however, seem to have evolved rather independently from one another since there is no phylogenetic correlation between the two. Chronic exposure to predation cues may result in either increased or decreased CORT levels depending on the species. Induced behavioural responses were largely disconnected from baseline CORT levels, while baseline CORT was negatively correlated with predator effects on morphology. Importantly, shifts in body shape caused by experimentally elevated CORT are distinct from predator-induced morphological responses, often affecting different aspects of body shape and sometimes showing even antagonistic morphological consequences. Thus, antipredator morphological responses seem to have evolved to be quite specific, and their trigger does not simply correspond to a general stress-response CORT increase. Moreover, predation cues have only minor effects on growth and developmental rate. Finally, we have observed a general pattern in which species with lower baseline CORT tend to have a lower CORT response to predation cues and a greater morphological response. We found that lower baseline CORT is also associated with slower development across species, and thus, as expected, longer larval period species tend to have a decreased CORT response to predation cues. However, we did not recover an association between larval period and morphological responses across species.

## Supplementary Material



## Data Availability

All the data used to perform the analyses and the commented R scripts can be found in the Zenodo repository [88]. Supplementary material is available online [89].
