# Supplementary material for: Evolutionary decoupling of morphological and behavioural antipredator responses and their hormonal basis across anuran species
Source: Proc Biol Sci. 2026 Oct 28;293(2081):20260887. doi: 10.1098/rspb.2026.0887 (PMC13725533; doi:10.1098/rspb.2026.0887)
Supplement: Supplementary file 1 [file rspb.2026.0887.zip › rspb.2026.0887/rspb20260887_si_001.pdf]

Supplementary Material

Evolutionary decoupling of morphological and behavioural antipredator responses and their hormonal basis across anuran species

Rafael Rico-Millan*, H. Christoph Liedtke, Lee Hyeun-Ji, Daniel R. Buchholz, Ivan Gomez-Mestre

*Corresponding author: rafael.rico@ebd.csic.es

Proceedings of the Royal Society B
DOI: https://doi.org/10.1098/rspb.2026.0887

Contents

Supplementary figures **3**
Figure S1 . . . 3
Figure S2 . . . 4
Figure S3 . . . 5
Figure S4 . . . 6
Figure S5 . . . 6
Figure S6 . . . 6

Supplementary Tables **7**

Experimental design **7**
Table S1 . . . 7

Endogenous CORT levels **7**
Table S2 . . . 7
Table S3 . . . 10
Table S4 . . . 10

Activity rate **13**
Table S5 . . . 13
Table S6 . . . 13
Table S7 . . . 13

Morphology **14**
Table S8 14
Table S9 15
Table S10 19
Table S11 20
Table S12 20

Metamorphosis **21**
Table S13 21
Table S14 22

Comparative phylogenetic analyses **23**
Table S15 23
Table S16 23
Table S17 24

Supplementary figures

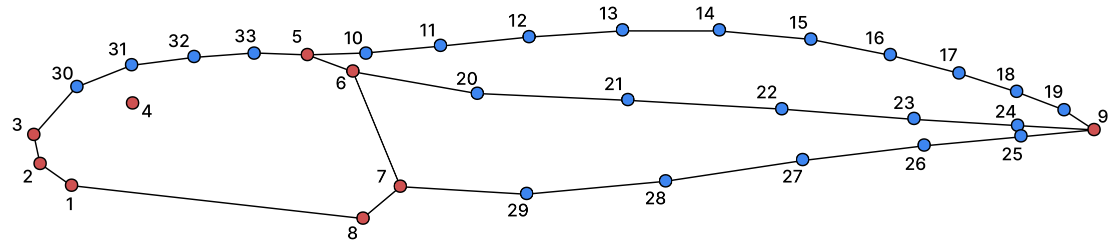

Landmarks

1: Lower lip
2: Upper lip
3: Snout tip
4: Eye
5: Dorsal insertion of tail fin
6: Dorsal body-tail muscle insertion
7: Ventral body-tail muscle insertion
8: Ventral insertion of tail fin
9: Tail tip

Semilandmarks

10-19: Dorsal edge of tail fin
20-24: Dorsal edge of tail muscle
25-29: Ventral edge of tail muscle
30-33: Dorsal edge of body

Figure S1. Landmark and semilandmark configuration used to capture tadpoles' shape geometric morphometric analyses. For X. laevis we changed the dorsal edge for the ventral edge of the tail.

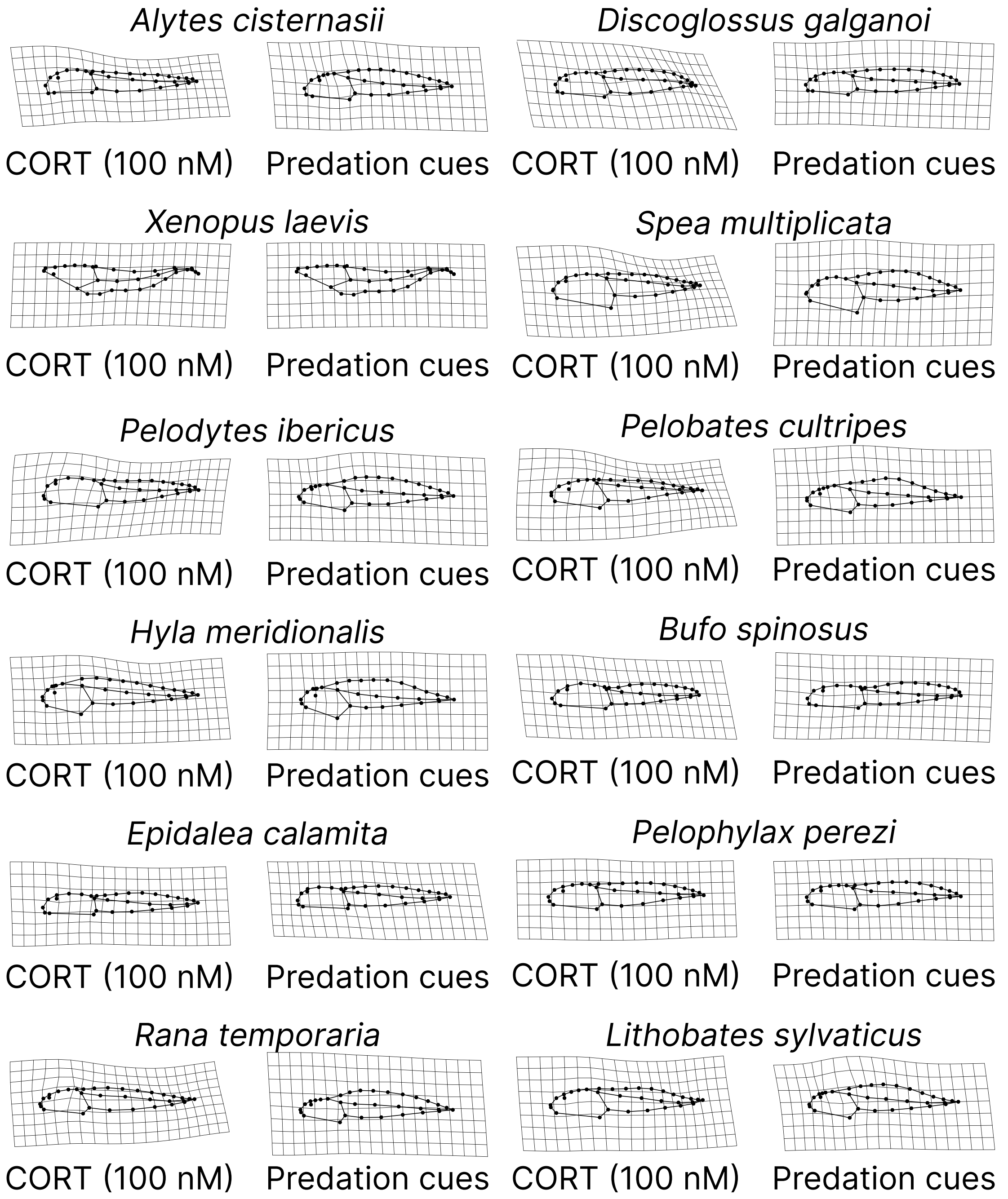

Figure S2. Mean shape and deformation grid of tadpoles of each species, exposed to 100nM CORT or predation cues compared to 0nM CORT or absence of predation cues respectively.

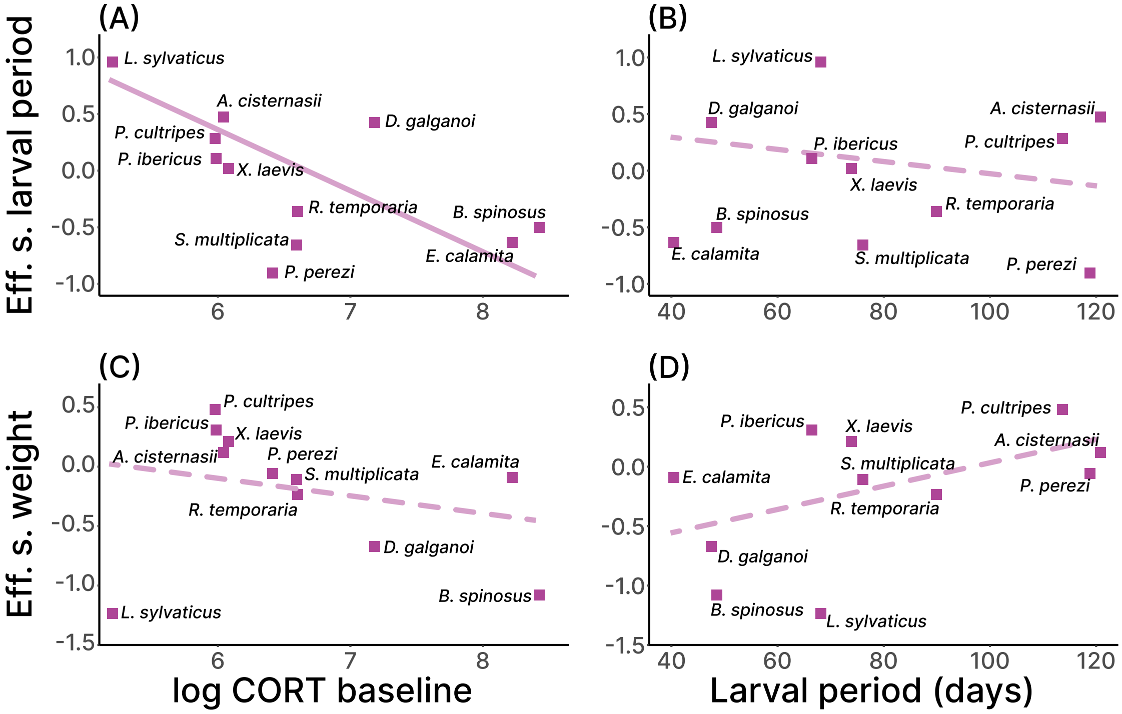

Figure S3. Relationship of endogenous CORT baseline levels and larval period length with the effect size (Hedges' G) of exposure to predation cues on (A and B) larval period length and (C and D) weight at metamorphosis. Lines show phylogenetic regression line, with solid and dashed lines indicating significant and non-significant relationships respectively, and purple squares indicate species values.

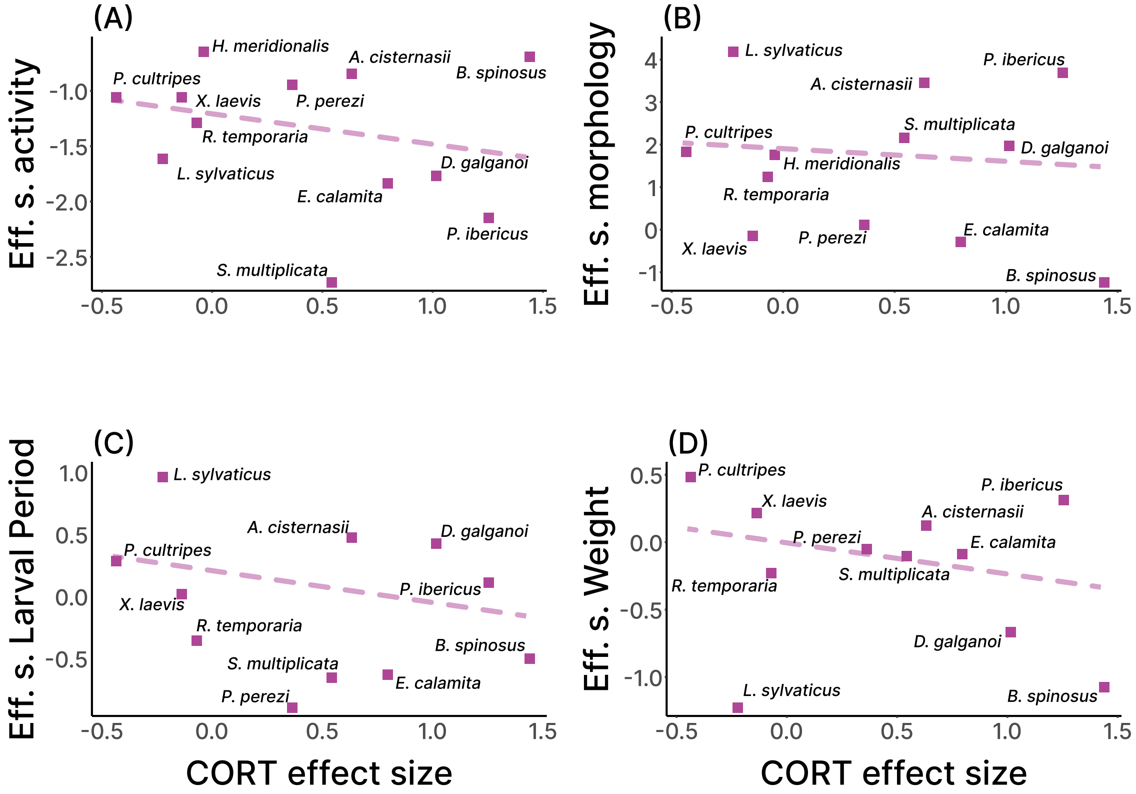

Figure S4. Relationship of changes in CORT levels with the effect size of exposure to predation cues on: (A) activity rate, (B) morphology, (C) larval period length, and (D) weight at metamorphosis. The effect sizes are Hedges' G except for morphology, where Z-values from the model are used instead. Solid and dashed regression lines indicate significant and non-significant relationships respectively (P-value < 0.05 as significance threshold).

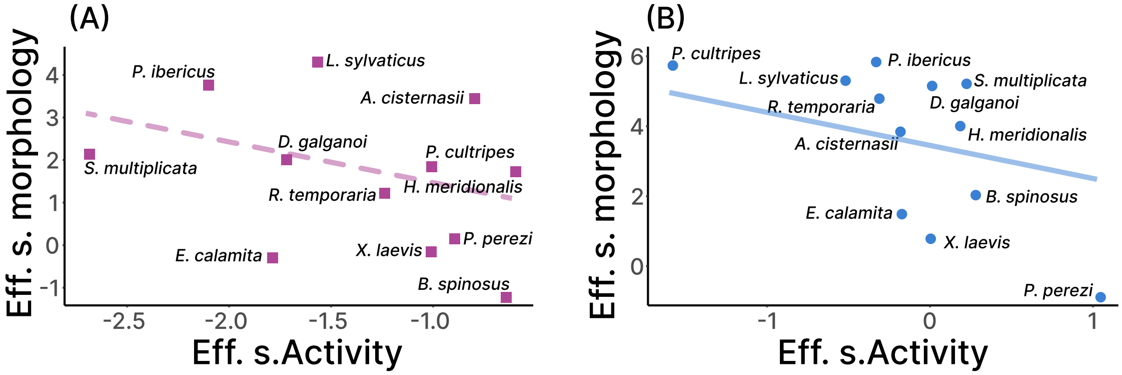

Figure S5. Relationship between effect sizes of exposure to predation cues (A) and exogenous CORT (B) on activity rate (Hedges' G) and morphology (Z-values). The lines show phylogenetic regression line, purple squares and blue circles indicate species values for exposure to predation cues and exogenous CORT respectively.

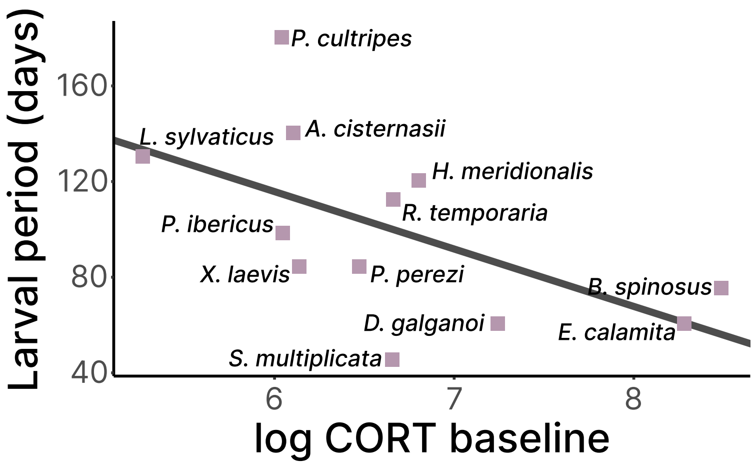

Figure S6. Relationship of endogenous CORT baseline levels and and larval period obtained from literature. The line shows the phylogenetic regression and the pink squares are the species values for larval period.

Supplementary Tables

Table S1: Sampling and experimental information of the studied species. Species flagged with * were obtained from captive breeding. Described are the developmental stage of sampled individuals, the number of ponds where they were sampled, the location of the ponds (for the captive bred species, the location of origin of the parents is given when known), and the predators used to generate the predation cues.

Species	Sampling description	Location	Predator
Alytes cisternasii	Tadpoles | 2 ponds	El Rocio (Huelva, Spain)	*Anax + Dytiscus*
Discoglossus galganoi	4 egg clutches | 2 ponds	Dos Hermanas (Sevilla, Spain)	*Anax + Dytiscus*
*Xenopus laevis**	3 egg clutches	Unknown	*Anax*
*Spea multiplicata**	4 egg clutches	Portal (Arizona, USA)	*Anax*
Pelodytes ibericus	5 egg clutches | 3 ponds	Castilblanco de los Arroyos (Sevilla, Spain)	*Anax*
Pelobates cultripes	4 egg clutches | 2 ponds	Cantillana (Sevilla, Spain)	*Anax + Dytiscus*
Hyla meridionalis	Tadpoles | 2 ponds	Doñana National Park (Huelva, Spain)	*Anax + Dytiscus*
Bufo spinosus	3 egg clutches | 1 pond	Cantillana (Sevilla, Spain)	*Anax + Dytiscus*
Epidalea calamita	3 egg clutches | 2 ponds	Puebla del Rio (Sevilla, Spain)	*Anax + Dytiscus*
Pelophylax perezi	Tadpoles | 1 pond	Dos Hermanas (Sevilla, Spain)	*Anax + Dytiscus*
Rana temporaria	3 egg clutches | 2 ponds	Llanes (Asturaias, Spain)	*Anax*
Lithobates sylvaticus	3 egg clutches | 1 pond	Anderson Township, Cincinnati (Ohio, USA)	*Anax*

Table S2: Summary of the linear models per species for the endogenous CORT level (Log transformed) of tadpoles exposed to control treatment (i.e. etanol), 50 nM CORT or 100 nM CORT. Significance levels: ***p<0.001, **p<0.01, *p<0.05, · p<0.1.

Species	Term	Estimate	Std Error	t value	Pr(>|t|)
Alytes cisternasii	(Intercept)	6.111	0.319	19.157	0.000 ***
	Treatment100	4.167	0.494	8.432	0.000 ***
	Treatment50	2.852	0.494	5.772	0.000 ***
Discoglossus galganoi	(Intercept)	7.573	0.125	60.745	0.000 ***
	Treatment100	3.338	0.176	18.931	0.000 ***
	Treatment50	2.723	0.169	16.130	0.000 ***
Xenopus laevis	(Intercept)	6.464	0.190	33.939	0.000 ***
	Treatment100	2.881	0.269	10.697	0.000 ***
	Treatment50	2.054	0.283	7.271	0.000 ***
Spea multiplicata	(Intercept)	7.285	0.363	20.064	0.000 ***
	Treatment100	1.737	0.480	3.616	0.002 **
	Treatment50	1.518	0.495	3.068	0.007 **
Pelodytes ibericus	(Intercept)	6.552	0.164	40.058	0.000 ***
	Treatment100	3.499	0.245	14.261	0.000 ***
	Treatment50	2.994	0.231	12.944	0.000 ***
Pelobates cultripes	(Intercept)	5.744	0.124	46.179	0.000 ***
	Treatment100	3.836	0.176	21.805	0.000 ***
	Treatment50	3.269	0.176	18.581	0.000 ***
Hyla meridionalis	(Intercept)	6.582	0.326	20.210	0.000 ***
	Treatment100	2.974	0.461	6.458	0.000 ***
	Treatment50	2.028	0.441	4.598	0.000 ***
Bufo spinosus	(Intercept)	9.031	0.181	49.919	0.000 ***
	Treatment100	2.422	0.256	9.466	0.000 ***
	Treatment50	1.706	0.256	6.670	0.000 ***
Epidalea calamita	(Intercept)	8.314	0.216	38.425	0.000 ***
	Treatment100	3.476	0.306	11.361	0.000 ***
	Treatment50	2.624	0.306	8.577	0.000 ***
Pelophylax perezi	(Intercept)	6.486	0.174	37.196	0.000 ***
	Treatment100	2.383	0.231	10.329	0.000 ***
	Treatment50	2.005	0.247	8.133	0.000 ***
Rana temporaria	(Intercept)	6.488	0.130	49.802	0.000 ***
	Treatment100	3.045	0.184	16.529	0.000 ***
	Treatment50	2.296	0.195	11.748	0.000 ***
Lithobates sylvaticus	(Intercept)	4.688	0.125	37.650	0.000 ***
	Treatment100	2.583	0.176	14.667	0.000 ***
	Treatment50	2.324	0.185	12.584	0.000 ***

Table S3: Tukey test for pairwise comparisons for endogenous CORT levels (Log transformed) of tadpoles from the exogenous CORT experiment. Significance levels: ***p<0.001, **p<0.01, *p<0.05, · p<0.1.

Species	Contrast	Difference	Lwr Conf	Upr Conf	Adj p value
Alytes cisternasii	100-0	4.167	2.873	5.460	0.000 ***
	50-0	2.852	1.559	4.146	0.000 ***
	50-100	-1.315	-2.711	0.082	0.066 .
Discoglossus galganoi	100-0	3.338	2.872	3.804	0.000 ***
	50-0	2.723	2.277	3.169	0.000 ***
	50-100	-0.615	-1.061	-0.169	0.008 **
Xenopus laevis	100-0	2.881	2.176	3.586	0.000 ***
	50-0	2.054	1.315	2.793	0.000 ***
	50-100	-0.827	-1.567	-0.088	0.028 *
Spea multiplicata	100-0	1.737	0.511	2.963	0.005 **
	50-0	1.518	0.255	2.781	0.017 *
	50-100	-0.219	-1.394	0.956	0.884
Pelodytes ibericus	100-0	3.499	2.836	4.161	0.000 ***
	50-0	2.994	2.369	3.619	0.000 ***
	50-100	-0.505	-1.167	0.158	0.145
Pelobates cultripes	100-0	3.836	3.366	4.305	0.000 ***
	50-0	3.269	2.799	3.738	0.000 ***
	50-100	-0.567	-1.036	-0.098	0.019 *
Hyla meridionalis	100-0	2.974	1.758	4.190	0.000 ***
	50-0	2.028	0.863	3.192	0.001 **
	50-100	-0.947	-2.111	0.218	0.119
Bufo spinosus	100-0	2.422	1.739	3.104	0.000 ***
	50-0	1.706	1.024	2.389	0.000 ***
	50-100	-0.715	-1.398	-0.033	0.040 *
Epidalea calamita	100-0	3.476	2.660	4.293	0.000 ***
	50-0	2.624	1.808	3.441	0.000 ***
	50-100	-0.852	-1.668	-0.036	0.041 *
Pelophylax perezi	100-0	2.383	1.791	2.974	0.000 ***
	50-0	2.005	1.373	2.638	0.000 ***
	50-100	-0.377	-0.969	0.215	0.259
Rana temporaria	100-0	3.045	2.547	3.543	0.000 ***
	50-0	2.296	1.768	2.823	0.000 ***
	50-100	-0.749	-1.277	-0.222	0.007 **
Lithobates sylvaticus	100-0	2.583	2.122	3.044	0.000 ***
	50-0	2.324	1.841	2.808	0.000 ***
	50-100	-0.259	-0.742	0.225	0.368

Table S4: Summary of the linear models for the endogenous CORT level (Log transformed) of tadpoles of different species exposed to control treatment (i.e. dechlorinated water) or predation cues. Significance levels: ***p<0.001, **p<0.01, *p<0.05, · p<0.1.

Species	Term	Estimate	Std Error	t value	Pr(>|t|)
Alytes cisternasii	(Intercept)	6.103	0.218	27.978	0.000 ***
	TreatmentP	0.444	0.308	1.440	0.167
Discoglossus galganoi	(Intercept)	7.242	0.171	42.354	0.000 ***
	TreatmentP	0.538	0.242	2.224	0.041 *
Xenopus laevis	(Intercept)	6.139	0.221	27.769	0.000 ***
	TreatmentP	-0.130	0.319	-0.409	0.686
Spea multiplicata	(Intercept)	6.656	0.187	35.600	0.000 ***
	TreatmentP	0.381	0.269	1.412	0.170
Pelodytes ibericus	(Intercept)	6.045	0.135	44.782	0.000 ***
	TreatmentP	0.576	0.191	3.019	0.007 **
Pelobates cultripes	(Intercept)	6.041	0.209	28.952	0.000 ***
	TreatmentP	-0.311	0.295	-1.054	0.306
Hyla meridionalis	(Intercept)	6.802	0.354	19.234	0.000 ***
	TreatmentP	-0.064	0.489	-0.130	0.898
Bufo spinosus	(Intercept)	8.487	0.141	60.030	0.000 ***
	TreatmentP	0.666	0.205	3.244	0.005 **
Epidalea calamita	(Intercept)	8.282	0.207	39.973	0.000 ***
	TreatmentP	0.506	0.279	1.810	0.087 .
Pelophylax perezi	(Intercept)	6.472	0.272	23.829	0.000 ***
	TreatmentP	0.338	0.384	0.879	0.389
Rana temporaria	(Intercept)	6.661	0.231	28.886	0.000 ***
	TreatmentP	-0.068	0.334	-0.203	0.841
Lithobates sylvaticus	(Intercept)	5.266	0.226	23.304	0.000 ***
	TreatmentP	-0.208	0.320	-0.651	0.521

Table S5: Summary of the linear models per species for activity rate (Arcsin transformed) of tadpoles exposed to control treatment (i.e. etanol), 50 nM CORT or 100 nM CORT. Significance levels: ***p<0.001, **p<0.01, *p<0.05, · p<0.1.

Species	Term	Estimate	Std Error	t value	Pr(>|t|)
Alytes cisternasii	(Intercept)	0.032	0.002	14.654	0.000 ***
	Treatment50	-0.005	0.003	-1.518	0.134
	Treatment100	-0.001	0.003	-0.196	0.845
Discoglossus galganoi	(Intercept)	0.043	0.003	13.140	0.000 ***
	Treatment50	0.001	0.005	0.314	0.754
	Treatment100	-0.002	0.005	-0.426	0.672
Xenopus laevis	(Intercept)	0.052	0.003	17.930	0.000 ***
	Treatment50	-0.001	0.004	-0.271	0.787
	Treatment100	-0.001	0.004	-0.254	0.801
Spea multiplicata	(Intercept)	0.094	0.002	60.160	0.000 ***
	Treatment50	0.002	0.002	0.988	0.327
	Treatment100	0.002	0.002	0.912	0.365
Pelodytes ibericus	(Intercept)	0.061	0.003	22.540	0.000 ***
	Treatment50	-0.002	0.004	-0.399	0.691
	Treatment100	-0.005	0.004	-1.355	0.180
Pelobates cultripes	(Intercept)	0.065	0.003	22.220	0.000 ***
	Treatment50	-0.017	0.004	-4.077	0.000 ***
	Treatment100	-0.026	0.004	-6.255	0.000 ***
Hyla meridionalis	(Intercept)	0.040	0.003	13.989	0.000 ***
	Treatment50	-0.001	0.004	-0.187	0.853
	Treatment100	0.002	0.004	0.449	0.655
Bufo spinosus	(Intercept)	0.017	0.002	7.842	0.000 ***
	Treatment50	0.003	0.003	1.107	0.273
	Treatment100	0.004	0.003	1.248	0.217
Epidalea calamita	(Intercept)	0.039	0.002	17.019	0.000 ***
	Treatment50	-0.008	0.003	-2.266	0.027 *
	Treatment100	-0.003	0.003	-0.927	0.358
Pelophylax perezi	(Intercept)	0.022	0.003	7.997	0.000 ***
	Treatment50	0.011	0.004	2.753	0.008 **
	Treatment100	0.011	0.004	2.819	0.006 **
Rana temporaria	(Intercept)	0.032	0.002	13.987	0.000 ***
	Treatment50	-0.002	0.003	-0.577	0.566
	Treatment100	-0.002	0.003	-0.615	0.540
Lithobates sylvaticus	(Intercept)	0.043	0.003	15.592	0.000 ***
	Treatment50	-0.009	0.004	-2.297	0.025 *
	Treatment100	-0.008	0.004	-2.014	0.048 *

Table S6: Tukey test for pairwise comparisons for activity rate (Arcsin transformed) of tadpoles from the exogenous CORT experiment. Significance levels: ***p<0.001, **p<0.01, *p<0.05, · p<0.1.

Species	Contrast	Difference	Lwr Conf	Upr Conf	Adj p value
Alytes cisternasii	50-0	-0.005	-0.012	0.003	0.289
	100-0	-0.001	-0.008	0.007	0.979
	100-50	0.004	-0.003	0.011	0.380
Discoglossus galganoi	50-0	0.001	-0.010	0.013	0.947
	100-0	-0.002	-0.013	0.009	0.905
	100-50	-0.003	-0.015	0.008	0.741
Xenopus laevis	50-0	-0.001	-0.011	0.009	0.960
	100-0	-0.001	-0.011	0.009	0.965
	100-50	0.000	-0.010	0.010	1.000
Spea multiplicata	50-0	0.002	-0.003	0.007	0.587
	100-0	0.002	-0.003	0.007	0.634
	100-50	0.000	-0.005	0.005	0.997
Pelodytes ibericus	50-0	-0.002	-0.011	0.008	0.916
	100-0	-0.005	-0.014	0.004	0.370
	100-50	-0.004	-0.013	0.006	0.607
Pelobates cultripes	50-0	-0.017	-0.027	-0.007	0.000 ***
	100-0	-0.026	-0.036	-0.016	0.000 ***
	100-50	-0.009	-0.019	0.001	0.082 .
Hyla meridionalis	50-0	-0.001	-0.010	0.009	0.981
	100-0	0.002	-0.008	0.012	0.895
	100-50	0.003	-0.007	0.012	0.799
Bufo spinosus	50-0	0.003	-0.004	0.010	0.514
	100-0	0.004	-0.003	0.011	0.430
	100-50	0.001	-0.006	0.007	0.982
Epidalea calamita	50-0	-0.008	-0.016	0.000	0.069 .
	100-0	-0.003	-0.011	0.005	0.626
	100-50	0.004	-0.004	0.013	0.430
Pelophylax perezi	50-0	0.011	0.001	0.020	0.020 *
	100-0	0.011	0.002	0.020	0.017 *
	100-50	0.000	-0.009	0.010	0.999
Rana temporaria	50-0	-0.002	-0.009	0.006	0.833
	100-0	-0.002	-0.010	0.006	0.812
	100-50	0.000	-0.008	0.008	0.999
Lithobates sylvaticus	50-0	-0.009	-0.018	0.000	0.063 .
	100-0	-0.008	-0.017	0.001	0.116
	100-50	0.001	-0.008	0.010	0.957

Table S7: Summary of the linear models per species for activity rate (Arcsin transformed) of tadpoles exposed to control treatment (i.e. dechlorated water) or predation cues. Significance levels: ***p<0.001, **p<0.01, *p<0.05, · p<0.1.

Species	Term	Estimate	Std Error	t value	Pr(>|t|)
Alytes cisternasii	(Intercept)	0.024	0.002	10.321	0.000 ***
	TreatmentP	-0.009	0.003	-2.780	0.007 **
Discoglossus galganoi	(Intercept)	0.053	0.003	17.276	0.000 ***
	TreatmentP	-0.029	0.004	-6.551	0.000 ***
Xenopus laevis	(Intercept)	0.050	0.002	21.574	0.000 ***
	TreatmentP	-0.013	0.003	-4.103	0.000 ***
Spea multiplicata	(Intercept)	0.094	0.003	35.333	0.000 ***
	TreatmentP	-0.034	0.004	-9.158	0.000 ***
Pelodytes ibericus	(Intercept)	0.062	0.002	29.887	0.000 ***
	TreatmentP	-0.025	0.003	-8.337	0.000 ***
Pelobates cultripes	(Intercept)	0.057	0.003	16.890	0.000 ***
	TreatmentP	-0.017	0.005	-3.729	0.000 ***
Hyla meridionalis	(Intercept)	0.046	0.003	13.660	0.000 ***
	TreatmentP	-0.011	0.005	-2.208	0.032 *
Bufo spinosus	(Intercept)	0.027	0.002	11.966	0.000 ***
	TreatmentP	-0.010	0.003	-3.055	0.004 **
Epidalea calamita	(Intercept)	0.042	0.002	21.940	0.000 ***
	TreatmentP	-0.023	0.003	-8.046	0.000 ***
Pelophylax perezi	(Intercept)	0.028	0.002	11.742	0.000 ***
	TreatmentP	-0.016	0.003	-4.801	0.000 ***
Rana temporaria	(Intercept)	0.031	0.002	14.332	0.000 ***
	TreatmentP	-0.020	0.003	-6.692	0.000 ***
Lithobates sylvaticus	(Intercept)	0.043	0.002	18.300	0.000 ***
	TreatmentP	-0.022	0.003	-6.695	0.000 ***

Table S8: Summary of the linear models using random permutation of residuals for the effect on morphology of tadpoles of different species exposed to different exogenous CORT or predation cues, controlling by allometric effects. Significance levels: ***p<0.001, **p<0.01, *p<0.05, · p<0.1.

	Df	Rsq	F value	Z value	P value
Treatment	4	0.007	8.737	6.248	0.001 **
species	10	0.119	57.062	16.698	0.001 **
log(Csize)	1	0.001	5.816	2.944	0.003 **
Treatment:species	40	0.042	5.064	13.796	0.001 **
Residuals	1299	0.27			
Total	1354				

Table S9: Pairwise comparisons for morphological changes of tadpoles induced by exogenous CORT and predation cues. Significance levels: ***p<0.001, **p<0.01, *p<0.05, · p<0.1.

Species	Comparison	d	UCL (95%)	Z value	P value
Alytes cisternasii	0:100	0.032	0.019	3.210	0.001 ***
	0:50	0.026	0.019	2.700	0.002 **
	0:NP	0.006	0.018	-1.866	0.969
	0:P	0.026	0.018	2.800	0.002 **
	100:50	0.008	0.018	-1.050	0.860
	100:NP	0.031	0.018	3.150	0.002 **
	100:P	0.049	0.018	4.936	0.001 ***
	50:NP	0.026	0.018	2.766	0.002 **
	50:P	0.045	0.017	4.490	0.001 ***
	NP:P	0.025	0.017	2.935	0.002 **
Discoglossus galganoi	0:100	0.043	0.018	4.229	0.001 ***
	0:50	0.030	0.017	3.276	0.001 ***
	0:NP	0.006	0.018	-1.984	0.980
	0:P	0.015	0.017	1.083	0.153
	100:50	0.017	0.018	1.420	0.084 .
	100:NP	0.043	0.018	4.327	0.001 ***
	100:P	0.043	0.018	4.389	0.001 ***
	50:NP	0.031	0.017	3.574	0.001 ***
	50:P	0.032	0.017	3.553	0.001 ***
	NP:P	0.017	0.017	1.563	0.056 .
Spea multiplicata	0:100	0.052	0.018	5.030	0.001 ***
	0:50	0.054	0.018	5.384	0.001 ***
	0:NP	0.010	0.017	-0.089	0.540
	0:P	0.020	0.017	2.118	0.019 *
	100:50	0.007	0.018	-1.177	0.882
	100:NP	0.059	0.017	5.674	0.001 ***
	100:P	0.064	0.017	6.148	0.001 ***
	50:NP	0.061	0.018	4.851	0.001 ***
	50:P	0.067	0.017	6.187	0.001 ***
	NP:P	0.017	0.016	1.883	0.030 *

Species	Comparison	d	UCL (95%)	Z value	P value
Pelodytes ibericus	0:100	0.045	0.018	4.701	0.001 ***
	0:50	0.044	0.018	4.454	0.001 ***
	0:NP	0.011	0.017	0.286	0.392
	0:P	0.020	0.017	2.216	0.013 *
	100:50	0.012	0.017	0.465	0.329
	100:NP	0.036	0.017	4.136	0.001 ***
	100:P	0.060	0.017	5.426	0.001 ***
	50:NP	0.034	0.018	3.622	0.001 ***
	50:P	0.056	0.018	4.556	0.001 ***
	NP:P	0.025	0.017	3.006	0.001 ***
Pelobates cultripes	0:100	0.053	0.017	5.330	0.001 ***
	0:50	0.053	0.017	5.436	0.001 ***
	0:NP	0.010	0.017	-0.061	0.524
	0:P	0.018	0.017	1.768	0.043 *
	100:50	0.006	0.018	-1.795	0.960
	100:NP	0.050	0.018	4.646	0.001 ***
	100:P	0.054	0.018	5.288	0.001 ***
	50:NP	0.050	0.018	4.477	0.001 ***
	50:P	0.054	0.017	6.394	0.001 ***
	NP:P	0.018	0.017	1.952	0.023 *
Hyla meridionalis	0:100	0.042	0.018	4.418	0.001 ***
	0:50	0.036	0.018	4.082	0.001 ***
	0:NP	0.016	0.018	1.274	0.092 .
	0:P	0.013	0.018	0.629	0.271
	100:50	0.011	0.018	0.115	0.445
	100:NP	0.031	0.018	3.567	0.001 ***
	100:P	0.043	0.018	4.177	0.001 ***
	50:NP	0.026	0.018	2.719	0.003 **
	50:P	0.037	0.017	3.994	0.001 ***
	NP:P	0.018	0.017	1.776	0.037 *

Species	Comparison	d	UCL (95%)	Z value	P value
Bufo spinosus	0:100	0.018	0.020	1.391	0.093 .
	0:50	0.014	0.019	0.722	0.238
	0:NP	0.013	0.019	0.503	0.320
	0:P	0.013	0.019	0.516	0.312
	100:50	0.009	0.018	-0.701	0.745
	100:NP	0.016	0.018	1.203	0.125
	100:P	0.018	0.017	1.802	0.039 *
	50:NP	0.015	0.018	1.187	0.110
	50:P	0.017	0.017	1.581	0.056 .
	NP:P	0.006	0.018	-1.617	0.944
Epidalea calamita	0:100	0.016	0.019	1.166	0.128
	0:50	0.018	0.019	1.417	0.085 .
	0:NP	0.010	0.017	-0.124	0.541
	0:P	0.007	0.018	-1.236	0.895
	100:50	0.010	0.021	-0.724	0.761
	100:NP	0.018	0.019	1.584	0.056 .
	100:P	0.018	0.019	1.366	0.084 .
	50:NP	0.021	0.018	2.085	0.022 *
	50:P	0.017	0.019	1.372	0.087 .
	NP:P	0.009	0.017	-0.356	0.646
Pelophylax perezi	0:100	0.008	0.018	-0.913	0.807
	0:50	0.008	0.017	-0.823	0.793
	0:NP	0.010	0.017	0.060	0.471
	0:P	0.013	0.016	0.733	0.241
	100:50	0.005	0.018	-2.351	0.990
	100:NP	0.011	0.017	0.278	0.389
	100:P	0.013	0.017	0.776	0.223
	50:NP	0.012	0.017	0.527	0.302
	50:P	0.015	0.017	1.262	0.111
	NP:P	0.010	0.017	0.190	0.422

Species	Comparison	d	UCL (95%)	Z value	P value
Rana temporaria	0:100	0.038	0.017	4.217	0.001 ***
	0:50	0.044	0.018	4.505	0.001 ***
	0:NP	0.012	0.017	0.437	0.331
	0:P	0.013	0.017	0.772	0.219
	100:50	0.017	0.018	1.592	0.065 .
	100:NP	0.037	0.017	4.355	0.001 ***
	100:P	0.047	0.018	4.804	0.001 ***
	50:NP	0.039	0.017	4.458	0.001 ***
	50:P	0.053	0.018	5.129	0.001 ***
	NP:P	0.018	0.017	1.845	0.037 *
Lithobates sylvaticus	0:100	0.048	0.018	4.917	0.001 ***
	0:50	0.038	0.018	4.093	0.001 ***
	0:NP	0.017	0.017	1.692	0.053 .
	0:P	0.023	0.017	2.402	0.005 **
	100:50	0.012	0.018	0.303	0.390
	100:NP	0.055	0.018	5.165	0.001 ***
	100:P	0.052	0.018	4.987	0.001 ***
	50:NP	0.045	0.017	4.513	0.001 ***
	50:P	0.045	0.018	4.621	0.001 ***
	NP:P	0.031	0.017	3.510	0.001 ***

Table S10: Results for the trajectory analyses per species, quantifying the trajectories of morphological change induced by exogenous CORT and predation cues. Shown are the path distance of each group, the magnitude differences and the trajectory correlation (i.e. angular differences) between the trajectories, along with their associated effect sizes and p-values. Significance levels: ***$p<0.001$, **$p<0.01$, *$p<0.05$, · $p<0.1$.

	Path distances		Magnitude difference			Trajectory correlation		
Species	PD CORT	PD Pred	d	Z (MD)	Pr > d	angle	Z (TC)	Pr > angle
Alytes cisternasii	0.029	0.024	0.005	0.832	0.209	120.851	1.362	0.092 .
Discoglossus galganoi	0.040	0.016	0.024	4.057	0.000 ***	90.662	0.022	0.497
Spea multiplicata	0.040	0.016	0.024	3.909	0.000 ***	107.210	0.777	0.239
Pelodytes ibericus	0.049	0.025	0.024	3.930	0.000 ***	155.182	2.792	0.000 ***
Pelobates cultripes	0.046	0.015	0.031	4.684	0.000 ***	115.736	1.126	0.142
Hyla meridionalis	0.031	0.015	0.016	2.888	0.001 ***	127.172	1.640	0.052 .
Bufo spinosus	0.019	0.007	0.012	2.237	0.009 **	112.277	0.983	0.175
Epidalea calamita	0.016	0.009	0.008	1.408	0.078 .	85.671	-0.172	0.563
Pelophylax perezi	0.008	0.009	0.002	-0.317	0.627	82.936	-0.301	0.610
Rana temporaria	0.037	0.013	0.023	3.889	0.000 ***	118.896	1.240	0.117
Lithobates sylvaticus	0.039	0.030	0.009	1.946	0.021 *	86.376	-0.142	0.557

Table S11: Summary of the linear models using random permutation of residuals for the effect on morphology of *X. laevis* tadpoles exposed to different exogenous CORT or predation cues, controlling by allometric effects. Significance levels: ***p<0.001, **p<0.01, *p<0.05, · p<0.1.

Species		Df	Rsq	F value	Z value	P value
Xenopus laevis	log(Csize)	1	0.1432	19.1807	4.15	0.001 **
	Treatment	4	0.0324	1.0851	0.4005	0.3516
	Residuals	110	0.8211			
	Total	115				

Table S12: Pairwise comparisons for morphological changes of *X.laevis* tadpoles induced by exogenous CORT and predation cues. Significance levels: ***p<0.001, **p<0.01, *p<0.05, · p<0.1.

Species	Comparison	d	UCL (95%)	Z value	P value
Xenopus laevis	0:100	0.021	0.030	0.863	0.207
	0:50	0.026	0.030	1.261	0.117
	0:NP	0.022	0.029	1.009	0.166
	0:P	0.016	0.031	0.238	0.398
	100:50	0.015	0.030	-0.060	0.517
	100:NP	0.010	0.027	-1.017	0.847
	100:P	0.016	0.029	0.159	0.442
	50:NP	0.017	0.029	0.384	0.340
	50:P	0.022	0.031	0.946	0.188
	NP:P	0.014	0.028	-0.078	0.522

Table S13: Summary of the linear models per species for larval period of tadpoles exposed to control treatment (i.e. dechlorated water) or predation cues. Significance levels: ***p<0.001, **p<0.01, *p<0.05, · p<0.1.

Species	Term	Estimate	Std Error	t Value	Pr(>|t|)
Alytes cisternasii	(Intercept)	120.154	1.680	71.525	0.000 ***
	TreatmentP	3.154	2.376	1.328	0.197
Discoglossus galganoi	(Intercept)	46.846	1.484	31.558	0.000 ***
	TreatmentP	2.517	2.193	1.148	0.263
Xenopus laevis	(Intercept)	73.214	0.988	74.091	0.000 ***
	TreatmentP	0.186	1.531	0.121	0.905
Spea multiplicata	(Intercept)	75.385	2.023	37.263	0.000 ***
	TreatmentP	-4.718	2.920	-1.616	0.120
Pelodytes ibericus	(Intercept)	65.786	0.675	97.395	0.000 ***
	TreatmentP	0.357	0.955	0.374	0.712
Pelobates cultripes	(Intercept)	113.000	2.735	41.310	0.000 ***
	TreatmentP	3.071	3.728	0.824	0.418
Bufo spinosus	(Intercept)	47.857	0.769	62.264	0.000 ***
	TreatmentP	-1.000	0.941	-1.062	0.301
Epidalea calamita	(Intercept)	39.833	0.711	56.033	0.000 ***
	TreatmentP	-1.548	1.171	-1.321	0.204
Pelophylax perezi	(Intercept)	118.143	4.018	29.403	0.000 ***
	TreatmentP	-9.743	5.239	-1.860	0.083 .
Rana temporaria	(Intercept)	89.286	1.866	47.842	0.000 ***
	TreatmentP	-2.357	2.639	-0.893	0.380
Lithobates sylvaticus	(Intercept)	67.429	1.563	43.153	0.000 ***
	TreatmentP	5.988	2.300	2.604	0.016 *

Table S14: Summary of the linear models per species for wheight at metamorphosis of tadpoles exposed to control treatment (i.e. dechlorated water) or predation cues. Significance levels: ***p<0.001, **p<0.01, *p<0.05, · p<0.1.

Species	Term	Estimate	Std Error	t Value	Pr(>|t|)
Alytes cisternasii	(Intercept)	0.395	0.012	33.458	0.000 ***
	TreatmentP	0.006	0.017	0.386	0.703
Discoglossus galganoi	(Intercept)	0.078	0.004	19.219	0.000 ***
	TreatmentP	-0.010	0.006	-1.624	0.119
Xenopus laevis	(Intercept)	0.378	0.024	15.956	0.000 ***
	TreatmentP	0.022	0.037	0.604	0.552
Spea multiplicata	(Intercept)	0.399	0.019	21.551	0.000 ***
	TreatmentP	-0.005	0.027	-0.197	0.846
Pelodytes ibericus	(Intercept)	0.254	0.008	32.966	0.000 ***
	TreatmentP	0.010	0.011	0.928	0.362
Pelobates cultripes	(Intercept)	2.323	0.173	13.464	0.000 ***
	TreatmentP	0.315	0.235	1.339	0.193
Bufo spinosus	(Intercept)	0.100	0.004	28.483	0.000 ***
	TreatmentP	-0.010	0.004	-2.362	0.029 *
Epidalea calamita	(Intercept)	0.077	0.004	21.092	0.000 ***
	TreatmentP	-0.001	0.006	-0.138	0.892
Pelophylax perezi	(Intercept)	0.410	0.028	14.480	0.000 ***
	TreatmentP	-0.002	0.037	-0.055	0.957
Rana temporaria	(Intercept)	0.270	0.012	22.176	0.000 ***
	TreatmentP	-0.010	0.017	-0.558	0.582
Lithobates sylvaticus	(Intercept)	0.252	0.009	27.785	0.000 ***
	TreatmentP	-0.042	0.013	-3.156	0.004 **

Table S15: Tests for phylogenetic signal using Blomberg's K and its associated P value, for the studied traits and trait plasticity of tadpoles exposed to predation cues (P) or to exogenous CORT (C). Significance levels: ***p<0.001, **p<0.01, *p<0.05, · p<0.1.

Trait	Blomsberg's K	P value
Larval period	0.491	0.391
Mean CORT level	0.666	0.130
Weight	0.572	0.255
Basal activity	0.728	0.063 .
CORT plasticity	0.415	0.606
Larval period plasticity	0.434	0.599
Weight plasticity	0.425	0.576
Activity plasticity (C)	0.463	0.514
Activity plasticity (P)	0.435	0.554
Morphology plasticity (C)	0.663	0.122
Morphology plasticity (P)	0.515	0.354

Table S16: Results for the phylogenetic linear models testing the relationship of the plasticity of studied traits with baseline corticosterone (CORT) level and larval period of tadpoles exposed to predation cues. Significance levels: ***p<0.001, **p<0.01, *p<0.05, · p<0.1.

Trait Response	Predictor	Adj R^2	b	Std.Error	t value	P value
CORT effect size	Baseline CORT	0.302	0.399	0.166	2.398	0.037 *
	Larval period	0.228	-0.013	0.006	-2.063	0.066 .
Activity effect size	Baseline CORT	-0.025	0.204	0.238	0.855	0.412
	Larval period	0.347	0.016	0.006	2.618	0.026 *
Morphology effect size	Baseline CORT	0.589	-1.631	0.399	-4.090	0.002 **
	Larval period	-0.074	-0.009	0.019	-0.496	0.630
Larval period effect size	Baseline CORT	0.439	-0.539	0.181	-2.971	0.016 *
	Larval period	-0.048	-0.005	0.007	-0.733	0.482
Weight effect size	Baseline CORT	-0.036	-0.147	0.182	-0.808	0.440
	Larval period	0.178	0.010	0.006	1.780	0.109

Table S17: Results for the phylogenetic linear models testing the relationship between the magnitude of change in studied traits and the change in endogenous CORT of tadpoles exposed to predation cues or to exogenous CORT. Significance levels: ***p<0.001, **p<0.01, *p<0.05, · p<0.1.

Experimental group	Trait	Adj R^2	b	Std Error	t value	P value
Predation cues	Larval period	-0.030	-0.256	0.305	-0.842	0.422
	Weight	-0.039	-0.231	0.291	-0.793	0.448
	Activity	-0.020	-0.274	0.310	-0.887	0.396
	Morphology	-0.084	-0.298	0.780	-0.381	0.711
Exogenous CORT	Activity	0.383	-0.145	0.052	-2.799	0.019 *
	Morphology	0.039	0.235	0.195	1.205	0.256
